# Inter-Day Reliability of Resting Metabolic Rate and Maximal Fat Oxidation during Exercise in Healthy Men Using the Ergostik Gas Analyzer

**DOI:** 10.3390/nu13124308

**Published:** 2021-11-29

**Authors:** Lidia Robles-González, Jorge Gutiérrez-Hellín, Millán Aguilar-Navarro, Carlos Ruiz-Moreno, Alejandro Muñoz, Juan Del-Coso, Jonatan R. Ruiz, Francisco J. Amaro-Gahete

**Affiliations:** 1 EFFECTS-262 Research Group, Department of Medical Physiology, Faculty of Medicine, University of Granada, 18011 Granada, Spain; lydiro@hotmail.es; 2 PROmoting FITness and Health through Physical Activity Research Group (PROFITH), Department of Physical and Sports Education, School of Sports Science, University of Granada, 18011 Granada, Spain; ruizj@ugr.es; 3 Exercise and Sport Science, Faculty of Health Sciences, Universidad Francisco de Vitoria, 28223 Pozuelo de Alarcón, Spain; jorge.gutierrez@ufv.es (J.G.-H.); millan.aguilar@ufv.es (M.A.-N.); alejandro.munoz@ufv.es (A.M.); 4 Exercise Physiology Laboratory, Camilo José Cela University, 28692 Villanueva de la Cañada, Spain; cruizm@ucjc.edu; 5 Centre for Sport Studies, Rey Juan Carlos University, 28943 Fuenlabrada, Spain; juan.delcoso@urjc.es

**Keywords:** reproducibility, RMR, RER, MFO, Fatmax, metabolic rate

## Abstract

The attainment of high inter-day reliability is crucial to determine changes in resting metabolic rate (RMR), respiratory exchange ratio (RER), maximal fat oxidation during exercise (MFO) and the intensity that elicits MFO (Fatmax) after an intervention. This study aimed to analyze the inter-day reliability of RMR, RER, MFO and Fatmax in healthy adults using the Ergostik gas analyzer. Fourteen healthy men (age: 24.4 ± 5.0 years, maximum oxygen uptake (VO_2_max): 47.5 ± 11.9 mL/kg/min) participated in a repeated-measures study. The study consisted of two identical experimental trials (Day 1 and Day 2) in which the participants underwent an indirect calorimetry assessment at resting and during an incremental exercise test. Stoichiometric equations were used to calculate energy expenditure and substrate oxidation rates. There were no significant differences when comparing RMR (1999.3 ± 273.9 vs. 1955.7 ± 362.6 kcal/day, *p* = 0.389), RER (0.87 ± 0.05 vs. 0.89 ± 0.05, *p* = 0.143), MFO (0.32 ± 0.20 vs. 0.31 ± 0.20 g/min, *p* = 0.776) and Fatmax (45.0 ± 8.6 vs. 46.4 ± 8.4% VO_2_max, *p* = 0.435) values in Day 1 vs. Day 2. The inter-day coefficient of variation for RMR, RER, MFO and Fatmax were 4.85 ± 5.48%, 3.22 ± 3.14%, 7.78 ± 5.51%, and 6.51 ± 8.04%, respectively. In summary, the current results show a good inter-day reliability when RMR, RER, MFO and Fatmax are determined in healthy men using the Ergostik gas analyzer.

## 1. Introduction

The assessment of the human resting metabolic rate (RMR) is considered of important relevance in both scientific and clinical settings, as it is a key parameter to determining caloric needs for energy balance and weight management [1,2]. To measure the effect of fasting, exercise, and nutritional interventions on RMR, it is crucial to have an instrument that is both reliable and accurate. The most extended method to determine RMR is indirect calorimetry, which uses oxygen uptake (VO_2_) and carbon dioxide production (VCO_2_) to estimate energy expenditure through the use of stoichiometry [3,4]. Substrate oxidation values (i.e., fat, carbohydrate) can also be calculated by indirect calorimetry using stoichiometric equations [4,5] based on respiratory gas exchange. In this regard, the respiratory exchange ratio (RER), obtained by dividing VCO_2_/VO_2_, is typically used to calculate fat and carbohydrate oxidation rates at rest and during exercise [6]. Although the collection of gas exchange data seems to be a valid method to assess all these metabolic variables, the use of different devices may introduce certain variability that hinders the measurement of changes induced by an exercise or nutrition intervention

In the market, there are several gas analyzers currently available to perform indirect calorimetry analysis. The Deltatrac Metabolic Monitor (VIASYS Health-care Inc., SensorMedics, Yorba Linda, CA, USA) is considered the gold standard method since it has a registered inter-day reliability lower than 4% for RMR [7]. On the other hand, the majority of commercial gas analyzers showed more than 10% inter-day reliability for RMR measurements [8,9]. There is a scientific consensus that these data are clinically unacceptable [7,10]. Given that the Deltatrac is no longer manufactured, no gold standard is recognized at the present time to determine not only RMR but also RER [11,12].

Over the last years, a growing interest has emerged in the concept of maximal fat oxidation during exercise (MFO), and the intensity that elicits MFO (Fatmax) as potential indicators of metabolic health and physical performance [13,14]. Therefore, it seems necessary to examine whether the procedures to determine MFO and Fatmax are reproducible in order to adequately interpret their clinical and practical importance [15]. Unclear results regarding inter-day reliability of MFO and Fatmax have been previously reported. While Chrzanowski-Smith et al. [15], Dandanell et al. [16] and Croci et al. [17] found a large within-subjects variation in MFO and Fatmax, De Souza Silveira et al. [18] and Marzouki et al. [19] observed a low coefficient of variation (CV [(Standard Deviation/Mean) × 100) for both MFO and Fatmax. Although these inconsistencies could be attributed to different factors (e.g., exercise protocols, ergometer type, or biological characteristics of the study participants), the gas analyzer used to register gas data collection seems to be an important source of variation [13].

The Ergostik gas analyzer (Geratherm Respiratory, Ergostik, Geratherm, Germany) has emerged as a promising device to collect indirect calorimetry related outcomes in both resting conditions (e.g., RMR or RER) and during exercise (e.g., MFO and Fatmax). This metabolic cart has a comfortable Ergoflow flowsensor (<20 g) and a powerful BLUE CHERRY^®^ diagnostic platform which provide the backbone for all testing, analysis and reporting. However, to the best of our knowledge, there is no study investigating the inter-day reliability of the above-mentioned outcomes using this untested breath-by-breath gas analyzer. Therefore, this study aimed to analyze the inter-day reliability of RMR, RER, MFO and Fatmax in healthy adults using the Ergostik gas analyzer.

## 2. Materials and Methods

### 2.1. Participants

A total of 14 healthy Caucasian men aged 19–33 years participated in the current study (Table 1). Inclusion criteria included: (i) being physically active (i.e., ~60 min of physical activity at least 4 days/week) over the last 6 months, (ii) not suffering any muscle-skeletal injury within the last 8 weeks, (iii) being non-smokers, (iv) not taking any drug or dietary supplement during the previous month, (v) not presenting any acute or chronic disease, and (vi) having a body mass index (BMI) lower than 25 kg/m^2^. The study protocol was conducted in accordance with the Declaration of Helsinki (last revision 2013) and approved by the Francisco de Vitoria University Research Ethics Committee (2020-18). The participants signed a written informed consent before their enrolment and were informed about the study procedures.

### 2.2. Procedures

The study was performed between October and December 2020. A repeated-measures design was implemented to obtain indirect calorimetry parameters. The participants attended our laboratory in two identical and matched trials (Day 1 and Day 2; 08:00–12:00 am) separated by 2 to 7 days at the same time. They fasted for >8 h before arrival to the laboratory on testing days, and were instructed to avoid vigorous physical activity during the previous 24 h. Similarly, a 24-h dietary register was obtained on Day 1 regarding the previous day, which was subsequently used to standardize the dietary pattern before the second testing day. Subjects were asked to record all food and beverages consumed from the time they awakened until the time they arrived in the laboratory the next day. They were also instructed to refrain from consuming alcohol, caffeine and stimulant substances during the 24 h prior to the trials.

On testing days, we conducted an indirect calorimetry assessment using a breath-by-breath gas analyzer (Ergostik, Geratherm Respiratory, Ergostik, Geratherm, Germany) at resting and during an incremental submaximal exercise test.

At rest, RMR was measured in accordance with the last revised guidelines of best practice for performing indirect calorimetry in healthy individuals [10]. Briefly, all measurements were conducted under controlled environmental conditions (temperature: 22–24 °C; humidity: 35–45%) in a quiet room by the same trained staff. Before the beginning of the RMR measurement, the participants rested in a supine position under thermoneutral conditions, breathing normally and not talking or fidgeting for at least 15 min. Similar instructions were provided during the RMR assessment period (i.e., 15 min). A 3-L syringe was employed to perform flow calibrations and two standard gas concentrations were used to conduct gas calibration (16.0% for O_2_; 5.0% for CO_2_) at the beginning of each trial. Obtained data from VO_2_, VCO_2_, RER and Ventilation (VE) were averaged every minute. Then, the CVs for each 5-min period were calculated after having discarded the first 5-min of data collection (i.e., 6th to 10th, 7th to 11th, 8th to 12th, 9th to 13th, 10th to 14th, and 11th to 15th). The 5-min periods meeting the steady state criteria (i.e., CV < 10% for VO_2_, VCO_2_, and VE, and CV < 5% for RER) [8,20] were subsequently identified. Finally, we considered for further analyses the 5-min period with the lowest average between CVs of VO_2_, VCO_2_, VE, and RER among the 5-min periods that met the steady state criteria. RMR was calculated from VO_2_, VCO_2_data applying the Weir stoichiometric equation [3] and RER value of this period was used for the statistical analysis.

After the RMR assessment (i.e., ~5 min), participants carried out a 10-min warm-up on a cycle ergometer (Ergoselect 4, Ergoline, Geratherm, Germany) at 30% of maximum oxygen uptake (VO_2_max). In Day 1, the participants were instructed to keep a cadence ranging from 70 to 90 rpm, which was replicated on Day 2. Exercise intensity was subsequently increased by 10% of VO_2_max every 3 min and the test finished when they registered a RER > 1. Gas exchange data were continuously registered using the above-mentioned breath-by-breath gas analyzer. Fat oxidation values were estimated from VO_2_ and VCO_2_ data averaged over the last minute of each 3-min stage [21], applying the Frayn stoichiometric equation [5]. We considered fat oxidation values as 0.0 g/min when RER > 1.0. MFO was recognized as the highest value of fat oxidation obtained during the submaximal exercise test. Fatmax was also registered as the intensity attained in the MFO stage.

One week before the first trial (Day 1), we organized an additional visit that involved an anthropometric and body composition analysis, and an incremental exercise test to determine VO_2_max. Similar pre-test instructions to those given in Day 1 and Day 2 were provided to the participants. Weight (kg) and height (cm) were assessed by a validated scale and stadiometer (Seca 700, Hammer Steindamm, Hamburg, Germany), and the BMI (kg/m^2^) calculated. Fat mass and fat free mass were estimated by bioimpedance (Tanita InnerScan Dual, RD-901BK36, Tokio, Japan). Afterwards, an incremental exercise test on a cycle ergometer was performed, which consisted of a 10-min warm-up at 50 W followed by increments of 25 W/min until volitional extenuation. The criteria for deeming VO_2_max to have been achieved were as follow: (a) to reach a steady in VO_2_ (i.e., increments lower than 2 mL/kg/min) despite an intensity increase, (b) to present a maximal heart rate between 10 bpm above or below the age-predicted maximum heart rate [20], and (iii) to attain a RER higher than 1.15 [22]. We considered as VO_2_max the highest VO_2_ value obtained over the last 1-min of the test when these criteria were not met. A regression analysis between wattage and VO_2_ was conducted for each participant to normalize exercise intensity in the experimental trials (Day 1 and Day 2) among all participants (i.e., increments of 10% VO_2_max).

### 2.3. Statistical Analysis

Raw gas exchange parameters (i.e., VO_2_, VCO_2_, VE, RMR, and RER) were downloaded to an Excel spreadsheet averaging them each minute. Then, their CVs were calculated for Day 1 and Day 2. Results are presented as means ± standard deviation unless otherwise stated. The assumption of normality was checked using a combination of visual inspection (i.e., histograms and scatter graphs) and statistical tests (i.e., Shapiro–Wilk test). We conducted the statistical analyses with the Statistical Package for Social Sciences (SPSS, v. 21.0, IBM SPSS Statistics, IBM Corporation) while graphs were plotted using GraphPad Prism 5 (GraphPad Software, San Diego, CA, USA). The level of significance was set to ≤ 0.05.

A two-sided paired *t*-test was performed to determine the absolute inter-day differences in RMR, RER, MFO and Fatmax values on Day 2 vs. Day 1. The Bland-Altman method [23] was also used to analyze inter-day reliability of the above-mentioned outcomes. In the Bland-Altman plots, Day 1 measurements were subtracted from Day 2 measurements, which implies that a positive value indicates that the results of Day 2 were higher than those obtained in Day 1. We also analyzed heteroscedasticity in order to study the error changes as a magnitude of measured changes.

## 3. Results

Table 1 shows the descriptive characteristics of the participants. No significant differences between participants were noted in energy and macronutrient intake (24 h before testing days) between participants.

**Figure 1:**
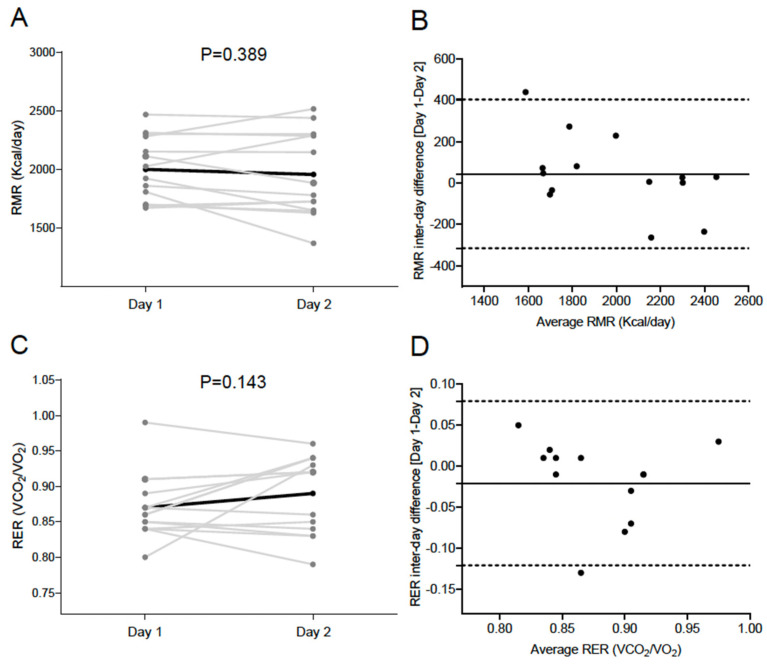
Comparison of Day 1 and Day 2 for resting metabolic rate (RMR) (**A**; kcal/day) and respiratory exchange ratio (RER) (**C**; carbon dioxide production [VCO_2_]/oxygen consumption [VO_2_]) in the study sample. The black line represents mean with individual data denoted by the grey lines. *p* value obtained by 2-sided paired *t*-tests. Bland–Altman plot displaying the difference in RMR (**B**) and RER (**D**) between Day 1 and 2. The solid line represents bias and the dashed lines represent lower and upper 95% limits of agreement.

A comparison of Day 1 and Day 2 for RMR and RER can be found in Figure 1. There were no significant differences when comparing both RMR (1999.3 ± 273.9 vs. 1955.7 ± 362.6 kcal/day, *p* = 0.389, Figure 1A) and RER (0.87 ± 0.05 vs. 0.89 ± 0.05, *p* = 0.143, Figure 1C) values in Day 1 vs. Day 2. Bland-Altman plots showed no systematic inter-day bias, with narrow limits of agreement in both RMR (Δ 43.7 [−315.9; 403.2] kcal/day, Figure 1B) and RER (Δ −0.02 [−0.12; 0.08], Figure 1D). No heteroscedasticity was detected either in RMR (β = −0.680; *p* = 0.180, Figure 1B) or in RER (β = 0.104; *p* = 0.475, Figure 1D). The inter-day CVs for RMR and RER were 4.85 ± 5.48% (43.7 ± 183.4 kcal/day) and 3.22 ± 3.14% (0.02 ± 0.05), respectively.

**Figure 2:**
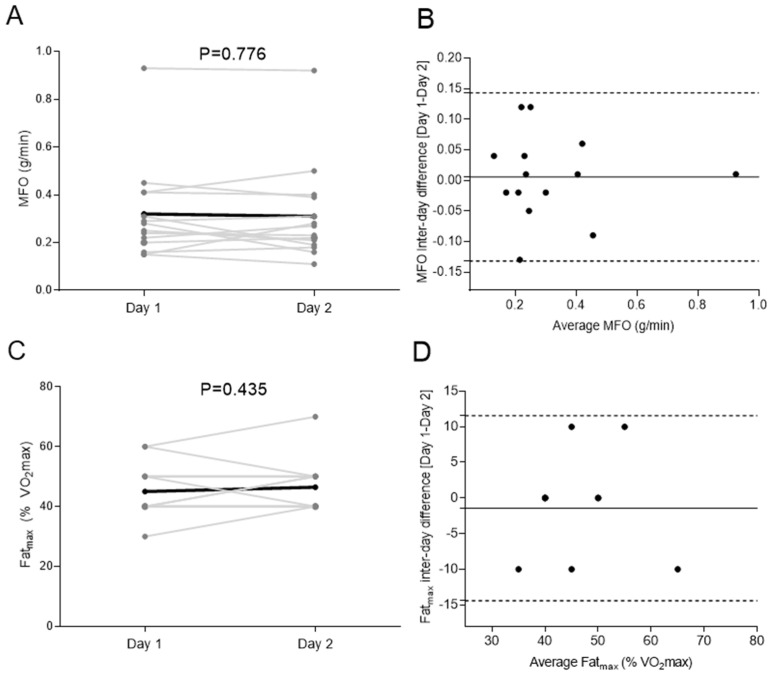
Comparison of Day 1 and Day 2 for maximal fat oxidation during exercise (MFO) (**A**; g/min) and the intensity that elicits MFO (Fatmax) (**C**; percentage of maximum oxygen uptake [VO_2_max]) in the study sample. The black line represents mean with individual data denoted by the grey lines. *p* value obtained by 2-sided paired *t*-tests. Bland–Altman plot displaying the difference in MFO (**B**) and Fatmax (**D**) between Day 1 and 2. The solid line represents bias and the dashed lines represent lower and upper 95% limits of agreement.

Figure 2 shows the comparison of Day 1 and Day 2 for MFO and Fatmax. No significant differences were observed when comparing both MFO (0.32 ± 0.20 vs. 0.31 ± 0.20 g/min, *p* = 0.776, Figure 2A) and Fatmax (45.0 ± 8.6 vs. 46.4 ± 8.4% VO_2_max, *p* = 0.435, Figure 2C) values in Day 1 vs. Day 2. Bland–Altman plots showed no systematic inter-day bias with narrow limits of agreement in both MFO (Δ 0.01 [−0.13; 0.14] g/min, Figure 2B) and Fatmax (Δ −0.02 [−0.12; 0.08]% VO_2_max, Figure 2D). No heteroscedasticity was detected neither in MFO (β = 0.134; *p* = 0.447, Figure 2B) nor in Fatmax (β = 0.072; *p* = 0.714, Figure 2D). The inter-day CVs for MFO and Fatmax were 7.78 ± 5.51% (0.01 ± 0.03 g/min) and 6.51 ± 8.04% (1.43 ± 3.57% VO_2_max), respectively.

## 4. Discussion

The main objective of the present study was to examine the inter-day reliability of RMR, RER at rest and MFO and Fatmax during exercise in healthy men using the Ergostik breath-by-breath gas analyzer. The overall results showed an acceptable inter-day reliability of RMR, RER, MFO and Fatmax in a homogeneous sample of healthy men, as evident by their reported dispersion. Moreover, there was no systematic bias when comparing RMR, RER, MFO and Fatmax Day 1 vs. Day 2 data. Taken together, the current findings confirm that the Ergostik is a reliable breath-by-breath gas analyzer to determine these metabolic outcomes and therefore, it may be consistently used to assess changes in metabolic variables induced by interventions.

Previous studies have highlighted the importance of achieving high RMR reliability in both cross-sectional and intervention studies [12,24]. Some studies investigating inter-day reliability of RMR have included mechanically ventilated patients obtaining a within-subject CV ranging from ~4% to ~10% and using the Ultima CardiO2 (Medgraphics Corp, Minnesota, MN, USA; CV~10%), the CCM Express (Medgraphics Corp, Minnesota, MN, USA; CV~8%), the Quark RMR (Cosmed, Italy; CV~4%) and the Deltatrac metabolic monitor (Datex-Ohmeda, Helsinki, Finland; CV~4%) [25]. On the other hand, few studies have been performed in healthy individuals [8,9,11,26]. Alcantara et al. and Sanchez-Delgado et al. reported an inter-day RMR CV ranging from ~13% to 19% in healthy sedentary adults using the Ultima CardiO2 and the CCM Express gas analyzers, respectively. Substantially low inter-day RMR CV values (i.e., CV~7%) were obtained by Haugen et al. assessing healthy individuals with the SensorMedics 2900 gas analyzer (SensorMedics Corp., Yorba Linda, CA, USA) using similar laboratory procedures. In addition, Cooper et al. investigated the reliability of six different gas analyzers, obtaining an inter-day RMR CV of ~11% for the Ultima CardiO2, ~11% for the Korr ReeVue (Korr Medical Technologies, Salt Lake City, UT, USA), ~8% for the Vmax Encore System (Viasys Healthcare, Inc., Yorba Linda, CA, USA), ~7% for MedGem (Microlife USA, Golden, CO, USA), ~5% for the TrueOne 2400 (Parvo Medics, Sandy, UT, USA) and ~4% for the Deltatrac metabolic monitor [11]. In our study, the inter-day CV for RMR was 4.85 ± 5.48% which is equivalent to 43.7 ± 183.4 kcal/day the Ergostik gas analyzer. This inter-day variability is similar to the one obtained with the gold standard method (i.e., Deltatrac metabolic monitor). Therefore, we can confirm that the Ergostik is a reliable breath-by-breath gas analyzer to determine this metabolic outcome.

Considerably less attention has been paid to the inter-day reliability of RER which also depends on VO_2_ and VCO_2_. In this regard, this variable is key when analyzing the amount of fat and carbohydrate rates oxidized at rest [27]. Previous studies have systematically reported poor inter-day reliability of RMR compared with RER in healthy individuals [8,9,11]. Cooper et al. [11] found no significant differences in RER inter-day reliability among five different gas analyzers and the Deltatrac metabolic monitor (CV < 5%). Similar CVs were also reported by Alcantara et al. [9] and Sanchez-Delgado et al. [8] when measuring RER at resting on different days. Interestingly, our results show even better inter-day RER reliability (CV = 3.2 ± 3.1%) than those obtained by the above-mentioned studies [8,9,11]. This would be due to relevant differences on the participant’ biological characteristics across studies since, while we recruited a homogeneous cohort of healthy men, heterogeneous samples were used in the others [8,9,11].

Currently, there is controversy regarding the inter-day reliability of MFO and Fatmax when measured during incremental exercise protocols. A recent study conducted by Chrzanowski-Smith et al. found that large inter-day variability is present when MFO (CV = 21%) and Fatmax (CV = 26%) are estimated through submaximal exercise test in healthy adults [15]. These findings partially agree with those obtained by Dandanell et al. [16] and Croci et al. [17] which reported an inter-day MFO CV of ~15% in individuals with obesity and recreationally trained men. However, considerably low MFO and Fatmax inter-day variability was reported by De Souza Silveira et al. [18] (CV = ~5% for MFO, and CV = ~7% for Fatmax) in recreational athletes of both sexes and by Marzouki et al. [19] (CV = ~3% for MFO) in sedentary subjects [17], which concur with those observed in our present study (CV = 7.7 ± 5.5% for MFO, and CV = 6.5 ± 8.0% for Fatmax). In all these investigations, the time between measurements was lower than seven days and all used incremental exercise protocols with 3 to 10 min stages, suggesting that these two characteristics were not responsible for the differences in reliability among investigations. These inconsistencies regarding the inter-day reliability of MFO and Fatmax could be explained by multiple factors, such as the ergometer type, the gas analyzer used, the exercise protocol, the data analysis approach or the fasting time/previous meal before the test [13]. Indeed, Croci et al. used the Douglas bag technique and a Servomex gas analyzer [17], whereas an Ergostik breath-by-breath gas analyzer was employed in our study. Therefore, further studies are needed to investigate congruent validity of MFO and Fatmax between different gas analyzer aiming to elucidate whether this specific factor plays a relevant role on inter-day reliability of MFO and Fatmax.

The present findings should be interpreted cautiously since some limitations are present. Firstly, our participants were healthy men and it remains unknown whether these results can be extended to women, older individuals or patients. Secondly, we do not know if the results apply to other gas analyzers given that we only used the Ergostik gas analyzer. Thirdly, we measured RMR for 15 min, which might be considered a too short register. However, considering that all participants achieved the 5-min steady state criteria, it should not be a limitation itself. Finally, MFO and Fatmax were obtained during an incremental exercise test and, therefore, we cannot confirm that inter-day reliability of these parameters would be similar than those obtained in response to a steady state exercise test. Further studies recruiting individuals with different biological characteristics than those included in the present work, and using additional gas analyzers, are needed to better understand the inter-day reliability of RMR, RER, MFO and Fatmax.

## 5. Conclusions

In summary, the current results demonstrate that low inter-day variability is present when RMR, RER, MFO and Fatmax were measured in a homogeneous cohort of healthy men using the Ergostik gas analyzer. Furthermore, there was no systematic bias in measures of RMR, RER, MFO and Fatmax across two matched testing trials. Collectively, our study findings confirm that the Ergostik is a reproducible gas analyzer to determine RMR, RER, MFO and Fatmax. Importantly, these conclusions should be extended when similar procedures to determine the above-mentioned parameters are applied in healthy men, and when the Ergostik gas analyzer is used to obtain indirect calorimetry data.

## Figures and Tables

**Table 1:** Characteristics of the study participants (*n* = 14).

Age (years)	24.4	±	5.0
Weight (kg)	74.1	±	13.8
Height (cm)	179.1	±	6.5
Body mass index (kg/m^2^)	22.9	±	2.8
Fat mass (%)	12.4	±	4.5
Lean mass (kg)	64.6	±	10.9
VO_2_max (L/min)	3.504	±	0.782
VO_2_max (mL/kg/min)	47.5	±	11.9

Values expressed as means ± standard deviation.

## Data Availability

Data from the present study are available upon reasonable request.
